# Cathepsin V suppresses GATA3 protein expression in luminal A breast cancer

**DOI:** 10.1186/s13058-020-01376-6

**Published:** 2020-12-09

**Authors:** Naphannop Sereesongsaeng, Sara H. McDowell, James F. Burrows, Christopher J. Scott, Roberta E. Burden

**Affiliations:** 1 School of Pharmacy, Medical Biology Centre, Queen’s University Belfast, 97 Lisburn Road, Belfast, BT9 7BL UK; 2 Patrick G Johnston Centre for Cancer Research, Medical Biology Centre, Queen’s University Belfast, 97 Lisburn Road, Belfast, BT9 7AE UK

**Keywords:** Cathepsin, Protease, Cancer, Invasion, GATA3, Luminal, Proliferation, Proteasome, Signalling

## Abstract

**Background:**

Lysosomal cysteine protease cathepsin V has previously been shown to exhibit elevated expression in breast cancer tissue and be associated with distant metastasis. Research has also identified that cathepsin V expression is elevated in tumour tissues from numerous other malignancies, but despite this, there has been limited examination of the function of this protease in cancer. Here we investigate the role of cathepsin V in breast cancer in order to delineate the molecular mechanisms by which this protease contributes to tumourigenesis.

**Methods:**

Lentiviral transductions were used to generate shRNA cell line models, with cell line validation undertaken using RQ-PCR and Western blotting. Phenotypic changes of tumour cell biology were examined using clonogenic and invasion assays. The relationship between GATA3 expression and cathepsin V was primarily analysed using Western blotting. Site-directed mutagenesis was used to generate catalytic mutant and shRNA-resistant constructs to confirm the role of cathepsin V in regulating GATA3 expression.

**Results:**

We have identified that elevated cathepsin V expression is associated with reduced survival in ER-positive breast cancers. Cathepsin V regulates the expression of GATA3 in ER-positive breast cancers, through promoting its degradation via the proteasome. We have determined that depletion of cathepsin V results in elevated pAkt-1 and reduced GSK-3β expression, which rescues GATA3 from proteasomal degradation.

**Conclusions:**

In this study, we have identified that cysteine protease cathepsin V can suppress GATA3 expression in ER-positive breast cancers by facilitating its turnover via the proteasome. Therefore, targeting cathepsin V may represent a potential therapeutic strategy in ER-positive breast cancers, by restoring GATA3 protein expression, which is associated with a more favourable clinical outcome.

**Supplementary Information:**

The online version contains supplementary material available at 10.1186/s13058-020-01376-6.

## Background

Cathepsin V (CTSV) is a human lysosomal cysteine protease, with normal physiological expression primarily found within the thymus, corneal epithelium and testis [1–3]. CTSV resides at human chromosome 9q22, the same loci as closely related family member, cathepsin L (CTSL) [3]. Studies have suggested that CTSV evolved from CTSL after mammalian divergence due to their high homology, but differences in their tissue distribution and active site morphology suggests that CTSV exerts distinct functions [4, 5]. CTSV has been shown to exhibit potent elastase activity [6], and its proteolytic activity has also been shown to be important for invariant chain processing in MHC-II antigen presentation within the thymus [7].

Whilst CTSV has not had the same extensive research to date as other cathepsin family members, there is increasing evidence associating this protease with multiple malignancies. Elevated expression of CTSV in tumour compared to normal tissue has been observed for squamous cell carcinoma, breast, colorectal and thymic epithelial cancers [2, 8–10], whereas more in depth analysis in hepatocellular and endometrial carcinomas has indicated that elevated CTSV expression is also associated with increasing tumour grade or stage [11, 12].

In relation to breast cancer, previous studies have also shown that CTSV expression is associated with distant metastasis [9]. Whilst these initial breast cancer studies did not clarify the particular subtype within which CTSV expression was elevated, CTSV has previously been associated with ER-positive breast cancers due to its inclusion on the Oncotype DX® genomic test. CTSV is one of 16 cancer related genes on the Oncotype DX® array which is used to assess the risk of ER-positive breast cancer recurrence and to determine the benefits of adjuvant chemotherapy treatment [13]. Further analysis of the Oncotype DX® gene signature by assessing copy number variation identified that CTSV was significantly associated with both reduced overall survival and disease-free survival in a cohort of breast cancer patients [14]. More recently, CTSV expression was also found to be elevated in breast ductal carcinoma in situ (DCIS), where it is associated with a poor outcome and has potential to predict DCIS progression to invasive disease [15].

GATA3 is a member of the zinc finger transcription factor family and has been shown to be essential for normal mammary gland development, with roles in essential processes such as luminal cell differentiation, adhesion and proliferation [16, 17]. Murine studies have shown that GATA3 expression is lost as luminal epithelial cells become less differentiated during breast cancer progression [18] and low GATA3 expression has been strongly associated with histological grade and positive lymph nodes, both of which are indicators of poor prognosis [19, 20].

GATA3 expression is highly correlated with ER-positive breast cancers and is associated with a favourable clinical outcome. In particular, highest expression levels of GATA3 have been observed in the luminal A subtype of ER-positive tumours [19, 21]. Numerous reports have detailed that loss of GATA3 expression is associated with a propensity for tumour metastasis, with mechanistic studies suggesting that GATA3 inhibits breast cancer metastasis by reversing epithelial-mesenchymal transition (EMT) [22, 23].

In this study, we investigate the role of CTSV in ER-positive breast cancer to determine the molecular mechanisms by which this protease contributes to tumourigenesis. We report that CTSV expression is associated with poor prognosis in breast cancer, particularly within the ER-positive subtype. Examination of CTSV shRNA cell line models reveals that CTSV facilitates proliferation and invasion of ER-positive breast cancer cells. We also show that CTSV can regulate the expression of GATA3 in ER-positive breast cancer cell lines. Collectively, these results indicate that CTSV is a prospective therapeutic target in ER-positive breast cancer.

## Methods

### Cell line culture and treatments

The human breast cancer cell lines MCF-7 and ZR-75-1 and 293 T human embryonic kidney cells were obtained from the American Type Culture Collection (ATCC). MCF-7 and 293 T cells were cultured in Dulbecco’s Modified Eagle Medium (DMEM) supplemented with 10% fetal calf serum (FCS) and 50 U/mL penicillin and 50 μg/ml streptomycin. ZR-75-1 cells were cultured in Roswell Park Memorial Institute-1640 media (RPMI-1640) supplemented with 1% sodium pyruvate, 10% fetal calf serum and 50 U/mL penicillin and 50 μg/ml streptomycin. All cells were grown at 37 °C in a humidified incubator with 5% CO_2_ and media were changed every 3 days. All cell culture media, supplements and antibiotics were purchased from Thermo Fisher Scientific. Hoechst 33258 (50 ng/mL) and actinomycin-D (5 μg/μl) were purchased from Thermo Fisher Scientific, whilst Bortezomib (100 nM) and Buparlisib (5 μM) were purchased from Insight Biotechnology. All were used according to manufacturer’s instructions.

### Lentiviral cell line generation and transient transfections

Lentiviral cell line generation was undertaken as previously described [24]. Briefly, 293 T cells were transfected with pLKO.1-shCTSV plasmids (Sigma-Aldrich), after which viral supernatant was harvested and used to transduce MCF-7 and ZR-75-1 cells. For transient transfections, cells were transfected using GeneJuice® transfection reagent in accordance to manufacturer’s instructions. pcDNA3.1-CTSV ORF (GeneScript), pCMV-Flag-STYX (MRC Protein Phosphorylation and Ubiquitylation Unit, University of Dundee) or empty vector control plasmids were transfected and incubated for 48 h before protein extraction and analysis. The catalytic cysteine residue of pcDNA3.1-CTSV ORF was mutated to a serine reside using site-directed mutagenesis (Agilent Technologies) to give rise to the mutant construct (mtCTSV). The shRNA-resistant plasmid (rCTSV) was also generated using site-directed mutagenesis of pcDNA3.1-CTSV ORF, with the targeting sequence of shCTSV-1 mutated to alter codon usage but still produce the same protein sequence.

### Western blotting

Whole cell lysates were prepared using RIPA buffer with quantification using the Pierce™ BCA Protein assay kit (ThermoFisher). Lysates were resolved by SDS-PAGE using 10% acrylamide gels and proteins were transferred onto polyvinylidene fluoride (PVDF) membrane by semi-dry blotting. The following antibodies were used in this study: goat polyclonal CTSV (BioTechne, AF1080), goat polyclonal GAPDH (BioTechne, AF5718), mouse monoclonal GATA3 (BioTechne, MAB6330), rabbit monoclonal pSer308 GATA3 (Abcam, ab186371), mouse monoclonal pThr308 Akt (BioTechne, MAB7419), rabbit polyclonal pSer473 Akt (Cell Signaling, 9271), mouse monoclonal Akt (BD Transduction Laboratories, P78020), rabbit polyclonal pSer9 GSK-3β (BioTechne, AF1590), mouse monoclonal GSK-3β (BioTechne, MAB25063) and mouse monoclonal FLAG M2 (Sigma-Aldrich, F3165). Secondary antibodies used were donkey anti-goat HRP conjugate (BioTechne, HAF109), goat anti-mouse HRP conjugate (BioRad, 172-1011) and goat anti-rabbit HRP conjugate (Cell Signaling, 70745). Protein visualisation was undertaken using Luminata™ Forte chemiluminescent detection reagent (Merck Millipore) and imaged using a Molecular Imager ChemiDoc XRS+ Imaging System (BioRad).

### RQ-PCR

RNA was extracted using Stat-60 (Amsbio) and cDNA prepared using the Transcriptor First Strand cDNA synthesis kit (Roche) according to manufacturer’s instructions. RQ-PCR was undertaken using the Lightcycler® 480 SYBR Green I Master reagent (Roche). Primer sequences used were as follows: CTSV-F: 5′-ggactctgaggaatcctatccat-3′, CTSV-R: 5′-gcaacagaattctcaggtctgtact-3′, GATA3-F: 5′-ctcattaagccccaagcgaag-3′, GATA3-R: 5′-tctgacagttcgcacaggac-3′, β-tubulin-F: 5′-cgcagaagaggaggaggatt-3′, β-tubulin-R: 5′-gaggaaaggggcagttgagt-3′. RQ-PCR was performed in duplicate for each gene investigated, with all experiments undertaken a minimum of 3 times. The fold change of mRNA expression was calculated by normalising absolute target gene expression levels to β-tubulin mRNA levels as an internal control. The average and standard deviation (SD) values were collated from three independent experiments and statistical analysis was determined by one-way-ANOVA using GraphPad Prism 8.

### Clonogenic assays

Clonogenic assays were performed by seeding optimised cell numbers in 6 well plates in triplicate and incubated at 37 °C in a humidified incubator with 5% CO_2_ for 11–13 days (cell-line dependent) to enable colony formation. Cells were fixed and stained with 0.4% crystal violet and the number of colonies containing at least 50 cells was determined in line with published protocols [25]. All assays were carried out in triplicate and colonies were counted using the Cell3 iMager neo. Results demonstrate the mean colony number per cell line ± SD. The average and SD values were collated from three independent experiments and statistical analysis was determined by one-way-ANOVA using GraphPad Prism 8.

### Invasion assays

Transwell inserts (Corning, UK) containing 8.0 μm polycarbonate membrane was coated with Matrigel (1 mg/mL) (BD Biosciences). Cells were seeded into the upper chamber in serum-free media, with growth media containing 10% FBS added to the lower chamber as a chemoattractant. Cells were incubated at 37 °C in a humidified incubator with 5% CO_2_ for 48 h. Invaded cells were fixed using Carnoy’s fixative and visualised by staining with Hoechst 33258 (50 ng/mL) prior to images being captured on a Leica DM5500 fluorescent microscope. Each cell line was analysed in triplicate with 9 images captured per membrane at × 20 magnification. Images were analysed using ImageJ and figures were generated using GraphPad Prism 8. The average and SD values representing the mean number of cells per field of view were collated from three independent experiments and statistical analysis was determined by one-way-ANOVA using GraphPad Prism 8.

### Extracellular ATP assay

Extracellular ATP was quantified by CellTiter-Glo® assay (Promega). Cells were seeded for 48 h before supernatants were collected for analysis. Fifty microliters of supernatants was added in triplicate to 96-well white tissue culture-treated plates (Corning) and mixed with 50 μL of CellTiter-Glo® reagent. Plates were agitated on a shaker in the dark for 5 min at 37 °C before luminescence was measured using a BioTek Synergy HT plate reader. Results were presented as the mean relative fold change and SD from three independent experiments, with statistical analysis determined by one-way-ANOVA using GraphPad Prism 8.

### Survival analysis

Online repository KM plotter (www.kmplot.com) was used to evaluate the correlation between CTSV mRNA expression and relapse free survival (RFS) [26]. Using the breast cancer dataset, survival dependent on CTSV expression was analysed based on ER status and intrinsic patient subtype (*N* = 3951) using a collation of previously published and publicly available Affymetrix microarray datasets, available through GEO, European Bioinformatics Institute and TCGA. Gene expression was evaluated using CTSV probe 210074_at, with patients split by best performing threshold and all data right censored at 120 months (10 years). Survival plots were presented as percentage survival versus time in months, with hazard ratio (HR), 95% confidence interval (CI) and log-rank *p* values calculated using GraphPad Prism 8.

### In silico analysis

The relationship between CTSV mRNA expression and GATA3 protein expression was assessed using cBioPortal (https://www.cbioportal.org) [27, 28], via examination of ER-positive breast cancers from the TCGA 2012 breast invasive carcinoma dataset. Two hundred ninety-eight samples within this cohort contained matched CTSV mRNA expression (microarray) and GATA3 protein expression (reverse phase protein array (RPPA)) and were subsequently used for analysis. Linear regression analysis and Spearman’s correlation were performed using GraphPad Prism 8.

## Results

### CTSV expression is associated with a poor survival in ER-positive breast cancers

**Fig. 1:**
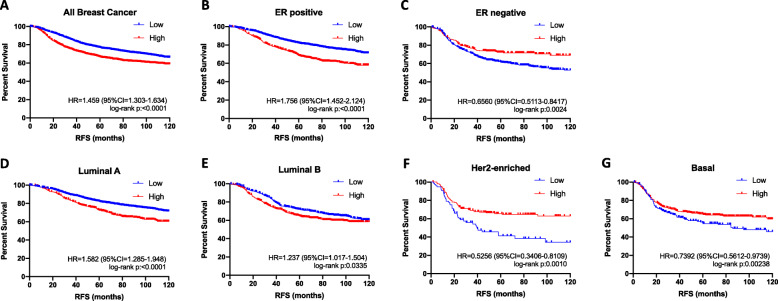
CTSV expression is associated with poor survival in ER-positive breast cancers. The relationship between CTSV mRNA expression and relapse free survival (RFS) was analysed using Kaplan-Meier survival curves across different breast cancer patient groups: **a** all breast cancer subtypes, **b** ER-positive, **c** ER-negative, **d** luminal A, **e** luminal B, **f** Her2-enriched and **g** basal. Results are expressed as percent survival relative to RFS in months. Hazard ratio (HR), 95% confident internals (95% CI) and log-rank *p* values are noted on each graph. All data was obtained from www.kmplot.com

To determine if there was a clinical association between CTSV expression and breast cancer, we assessed gene expression and survival analysis using KM Plot (www.kmplot.com). Examination of all breast cancer cases (*N* = 3951) identified that high CTSV expression was associated with poor prognosis (HR = 1.459, log-rank *p* = < 0.0001) (Fig. 1a). Given the inclusion of CTSV on the Oncotype DX® array for predicting ER-positive breast cancer recurrence, we then examined the impact of CTSV expression on breast tumours dependent on ER expression. Examination of ER-positive versus ER-negative breast cancers highlighted clear opposing effects, with high CTSV expression associated with poor prognosis in ER-positive tumours (*N* = 2061) (HR = 1.756, log-rank *p* = < 0.0001), but correlating with a more favourable outcome in ER-negative patients (*N* = 801) (HR = 0.656, log-rank *p* = 0.0024) (Fig. 1b, c). More detailed analysis was also undertaken by examining the impact of CTSV on survival based upon stratification into the 4 classical breast cancer intrinsic subtypes: luminal A, luminal B (both ER-positive), Her2-enriched and basal (both ER negative). Examination of cases classified into luminal A (*N* = 1933) and luminal B (*N* = 1149) subtypes identified that elevated CTSV expression was associated with poor prognosis in both subtypes, with the significance much greater in luminal A tumours (HR = 1.582, log-rank *p* = < 0.0001) compared to luminal B tumours (HR = 1.237, log-rank *p* = 0.0335) (Fig. 1d, e). In contrast, analysis of Her2-enriched (*N* = 251) (HR = 0.5256, log-rank *p* = 0.0010) and basal tumours (*N* = 618) (HR = 0.7392, log-rank *p* = 0.00238) revealed that elevated CTSV expression was associated with a more favourable outcome, in agreement with that observed when ER-negative patients were analysed collectively (Fig. 1f, g).

### CTSV regulates proliferation and invasion in ER-positive breast cancer

**Fig. 2:**
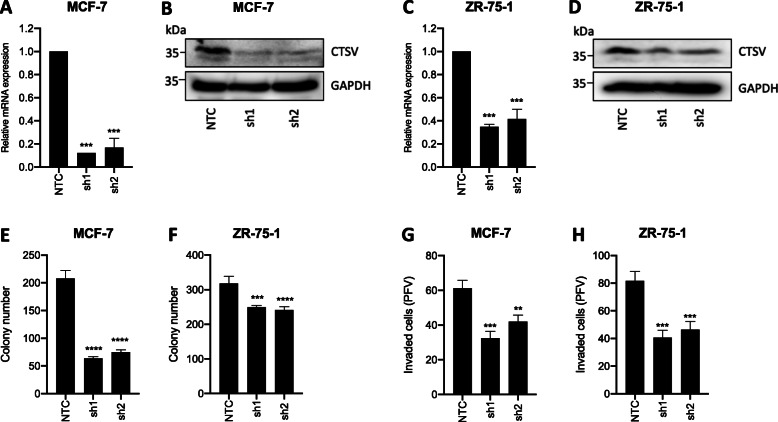
CTSV drives ER-positive breast cancer cell proliferation and invasion. MCF-7 and ZR-75-1 shCTSV-expressing cell line models (sh1 and sh2) were generated and validated by **a**, **c** RQ-PCR and **b**, **d** Western blotting. GAPDH was used as an internal loading control for Western blotting analysis. **e**, **f** The impact of CTSV expression on cell proliferation was assessed by clonogenic assay and **g**, **h** invasion assay. Average and SD values presented are collated from 3 independent experiments, **p < 0.01, ***p < 0.001, ****p < 0.0001

In order to examine the potential role of CTSV in ER-positive breast cancer, focusing on the luminal A subtype due to the stronger association between CTSV expression and survival, genetic ablation models were developed in MCF-7 and ZR-75-1 cells using CTSV-targeting shRNAs. Successful depletion of CTSV expression using two independent CTSV-targeting shRNA sequences was verified using RQ-PCR and Western blotting, with comparisons made to cells transduced with a non-targeting control shRNA (NTC) (Fig. 2a–d).

The impact of CTSV on cell proliferation was subsequently assessed by clonogenic assay, where CTSV depletion resulted in a significant impairment in colony formation in both MCF-7 and ZR-75-1 cell line models (Fig. 2e, f). Furthermore, given the classical role of cysteine proteases in mediating tumour cell invasion, in vitro invasion through Matrigel was also examined. As expected, CTSV silencing by shRNA led to significantly reduced tumour cell invasion compared to NTC cells (Fig. 2g, h). Representative images from the clonogenic and invasion assays are illustrated in Supplementary Fig. 1.

### CTSV expression is inversely correlated with GATA3 in ER-positive breast cancer

**Fig. 3:**
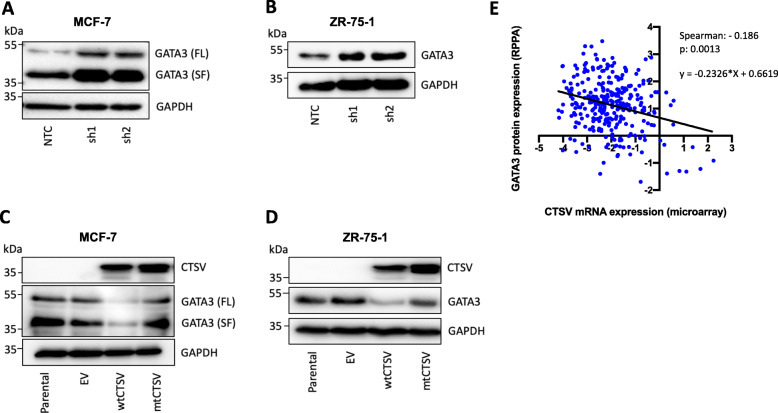
CTSV expression is inversely correlated with GATA3 expression. **a**, **b** Western blotting analysis of GATA3 protein expression in MCF-7 and ZR-75-1 shCTSV line models. **c**, **d** Western blotting analysis of GATA3 expression in MCF-7 and ZR-75-1 cells after overexpression of wildtype CTSV (wtCTSV) or a catalytic cysteine mutant CTSV (mtCTSV). GAPDH was used as an internal loading control for all Western blotting analysis. **g** Linear regression analysis and Spearman’s correlation between GATA3 protein expression (RPPA) and CTSV mRNA expression (microarray) in ER-positive breast patients from the TCGA 2012 breast invasive carcinoma dataset (cBioPortal)

Western blotting analysis identified an inverse correlation between CTSV and GATA3 protein expression in both MCF-7 and ZR-75-1 cells, where depletion of CTSV expression by shRNA resulted in a notable increase in GATA3 protein levels (Fig. 3a, b, Supplementary Fig. 2A). Confirmation of this relationship was observed following CTSV overexpression (wildtype CTSV = wtCTSV), where GATA3 expression was markedly diminished. Interestingly, overexpression of a catalytic mutant CTSV (mtCTSV), where the active site cysteine residue was mutated to a serine residue, had little impact on GATA3 protein levels in comparison to control cells, suggesting that the mechanism by which CTSV regulates GATA3 was largely dependent on its proteolytic activity (Fig. 3c, d, Supplementary Fig. 2B). It was also noted that the GATA3 splice form (SF) present within MCF-7 cells due to a heterozygous frameshift mutation, was also altered by CTSV, suggesting that the mechanism of regulation was not restricted to the wildtype protein.

In silico analysis of the relationship between CTSV mRNA expression and GATA3 protein expression was also examined in clinical samples, using the TCGA 2012 breast invasive carcinoma dataset via cBioPortal [27, 28]. GATA3 protein expression, as determined by RPPA analysis, was found to be statistically correlated with CTSV expression (p: 0.0013). Patients exhibiting high CTSV expression had notably less GATA3 protein expression than patients with low CTSV expression (Fig. 3e).

### CTSV regulates GATA3 via proteasomal degradation

**Fig. 4:**
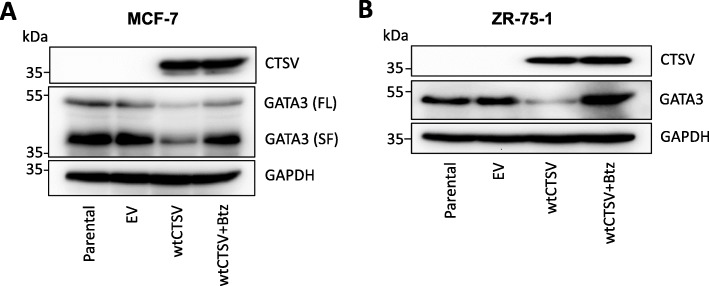
CTSV regulation of GATA3 is mediated via proteasomal degradation. **a**, **b** Western blotting analysis of GATA3 expression in MCF-7 and ZR-75-1 cells following overexpression of wildtype CTSV (wtCTSV) in combination with Bortezomib (Btz) treatment (100 nM for 6 h) to inhibit the proteasome. GAPDH was used as an internal loading control for Western blotting analysis

Numerous studies have examined factors which regulate the expression of GATA3, with mechanisms ranging from transcriptional regulation, RNA stability and posttranslational control [29–31]. RQ-PCR analysis and experiments with actinomycin D treatment (5 μg/μl for 8 h), suggested that CTSV regulation of GATA3 was not mediated via transcription or RNA stability (Supplementary Fig. 3). Therefore, to examine the potential for CTSV to exert posttranslational regulation of GATA3, cells were transfected with the wtCTSV overexpression construct and treated with proteasome inhibitor, Bortezomib (100 nM for 6 h). Analysis by Western blot confirmed that CTSV regulation of GATA3 was mediated via the proteasome; overexpression of CTSV resulted in reduced GATA3 protein expression, which was subsequently rescued following Bortezomib treatment (Fig. 4a, b, Supplementary Fig. 4).

### CTSV regulates GATA3 phosphorylation via Akt-1 and GSK-3β

**Fig. 5:**
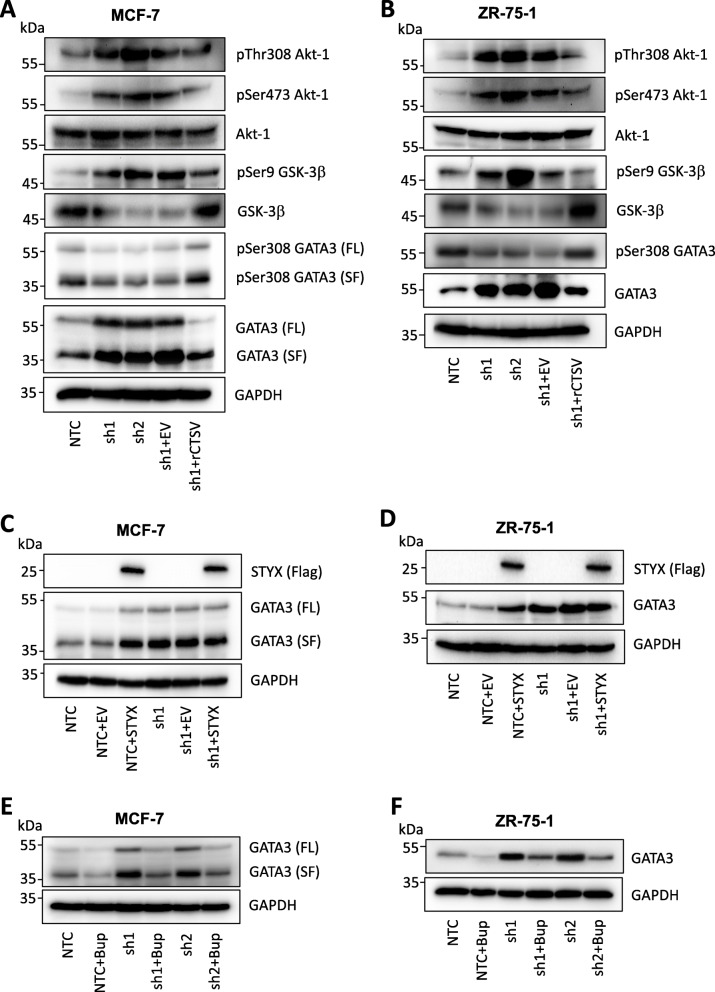
CTSV mediates GATA3 degradation through Akt and GSK-3β signalling. **a**, **b** Western blotting analysis of pThr 308 Akt, pSer473 Akt, Akt, pSer9 GSK-3β, GSK-3β pSer308 GATA3 and GATA3 expression in MCF-7 and ZR-75-1 shCTSV cell line models. Restoration of CTSV expression in sh1 cells was enabled by the generation and transfection of an shRNA-resistant rescue construct (rCTSV). **c**, **d** Western blotting analysis of GATA3 expression following transfection of STYX (Flag-tagged) or pCMV empty vector (EV) into MCF-7 or ZR-75-1 cells. **e**, **f** Western blotting analysis of GATA3 expression in MCF-7 and ZR-75-1 cells following treatment with Buparlisib (250 μM, 6 h). GAPDH was used as an internal loading control for all Western blotting analysis

To dissect the molecular mechanisms by which CTSV regulates the proteasomal turnover of GATA3, we examined signalling pathways previously associated with GATA3 regulation. Cells with depleted CTSV expression were found to exhibit activation of the Akt pathway, identified by increased expression of pThr308 and pSer473 Akt-1, but with no discernable impact on total Akt-1 expression. To confirm these findings, a sh1-resistant CTSV overexpression rescue construct (rCTSV) was generated by site-directed mutagenesis. Transfection of pcDNA3.1 empty vector (EV) into sh1 cells had no impact on Akt-1 phosphorylation, whereas transfection of the rescue construct (rCTSV) led to a decrease in both pThr308 and pSer473 Akt-1 phosphorylation (Fig. 5a, b, Supplementary Fig. 5A and B).

Previous studies have shown that Akt inhibits GSK-3β activity by phosphorylating serine residue 9 [31]. This inhibitory phosphorylation leads to a reduction in GSK-3β protein expression, due to degradation by the proteasome [32]. Examination of shCTSV cells revealed that pSer9 GSK-3β levels were elevated and total GSK-3β expression was reduced in comparison to NTC cells. Transfection of rCTSV led to the reduction of pSer9 GSK-3β and increase of total GSK-3β expression, confirming the involvement of CTSV (Fig. 5a, b, Supplementary Fig. 5A and B). Phosphorylation of GATA3 at residue Ser308 has previously been used as a marker of proteasomal turnover in ER-positive breast cancer cells [29]. Western blotting analysis reveals a notable reduction of pSer308 GATA3 in CTSV depleted cells, correlating with increased total GATA3 protein expression, whereas rCTSV expression restores pSer308 and total GATA3 expression to that observed in NTC cells (Fig. 5a, b, Supplementary Fig. 5A and B).

GSK-3β has previously been shown to phosphorylate GATA3, enabling recognition of GATA3 by the E3 ligase F-box protein FBXW7α. FBXW7α has been shown to specifically interact with GATA3, targeting it for degradation, based upon its phosphorylation by GSK-3β in MCF-7 cells. Therefore, to confirm the involvement of FBXW7α in CTSV-mediated degradation of GATA3, transfection of a construct encoding the pseudophosphatase STYX, which has previously been shown to inhibit FBXW7α, was undertaken [33]. Whilst transfection of the pCMV empty vector (EV) into NTC cells had no impact on GATA3 protein levels, introduction of STYX led to a notable increase in GATA3 expression levels, to levels similar to that observed in the shCTSV cells. Furthermore, transfection of EV or STYX into the shCTSV cells had no additional impact on GATA3 expression levels, suggesting that CTSV mediates GATA3 proteasomal degradation via FBXW7α (Fig. 5c, d, Supplementary Fig. 5C).

**Fig. 6:**
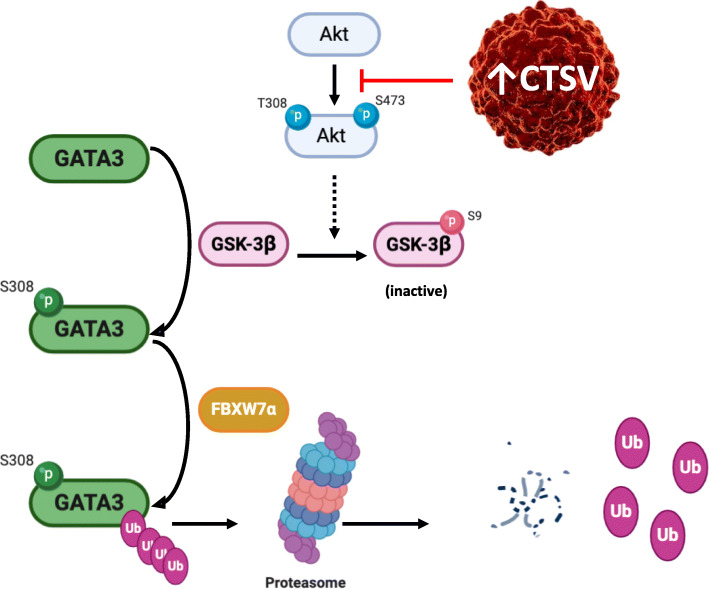
Proposed molecular mechanism by which CTSV regulates GATA3 protein expression in ER-positive breast cancer. Figure was created with BioRender.com

To examine this pathway in more detail, and determine if CTSV acts upstream of Akt, MCF-7 and ZR-75-1 cells were treated with PI3K inhibitor, Buparlisib (250 μM for 6 h). In line with previous studies examining the impact of PI3K on GATA3 protein expression [34], treatment of shCTSV cells with Buparlisib resulted in a dramatic reduction in the elevated GATA3 protein expression associated with CTSV depleted cells (Fig. 5e, f, Supplementary Fig. 5D and E). Collectively, these results suggest that CTSV functions upstream of Akt to negatively regulate GATA3 protein expression, via the Akt-GSK-3β-FBXW7α pathway, as illustrated in Fig. 6.

### CTSV regulates extracellular ATP levels

**Fig. 7:**
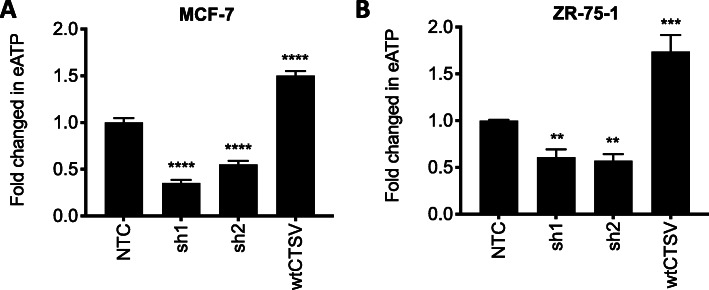
CTSV depletion results in attenuated eATP levels. **a**, **b** eATP was measured in supernatants from MCF-7 and ZR-75-1 shCTSV cell line models using the CellTiter Glo ATP assay. Data is presented as mean relative fold change in eATP from 3 independent experiments with standard deviation, **p < 0.01, ***p < 0.001, ****p < 0.0001

A recent study by Li and colleagues identified that GATA3 could reduce extracellular ATP (eATP) in the breast cancer microenvironment by upregulating ectonucleoside triphosphate diphosphohydrolase 3 (ENTPD3), an eATP hydrolytic enzyme [35]. Elevated eATP in the tumour microenvironment has been shown to facilitate EMT, cell migration and metastasis, with GATA3 functioning in an anti-metastatic manner by promoting eATP hydrolysis. To determine the impact of CTSV, we measured eATP in the supernatant from CTSV shRNA and overexpression cell line models. As anticipated, eATP levels were significantly reduced in CTSV depleted cells, whereas cells overexpressing wtCTSV displayed significantly enhanced eATP levels. Similar results were observed in both MCF-7 and ZR-75-1 models (Fig. 7a, b).

## Discussion

The work presented here examines for the first time the relationship between CTSV expression and relapse free survival of breast cancer patients. We have shown that CTSV expression has distinctly different outcomes across breast cancer subtypes, particularly in relation to the ER status of the tumour. This would suggest that CTSV may have distinctive roles across breast cancer subtypes; dissecting these roles would require extensive additional research using models representing the different breast cancer subtypes. Given that elevated CTSV expression was associated with reduced survival in ER-positive tumours, we wanted to determine if CTSV may represent a future therapeutic target in this subtype of the disease. Therefore, the research presented herein has focused on dissecting the mechanism of CTSV in ER-positive breast cancer, utilising cell line models representative of the luminal A subtype due to the greater impact of CTSV expression in relation to patient RFS.

Endocrine therapies developed for ER-positive breast cancers such as Tamoxifen, Fulvestrant and aromatase inhibitors such as Anastrozole and Exemestane have exhibited significant clinical success [36]. However, 30–50% patients treated with these endocrine therapies subsequently develop resistance and disease recurrence can occur up to 20 years post-diagnosis, with recurrence rates of 10–41% dependent on tumour stage at diagnosis and nodal involvement [37]. Whilst only approximately 10% of ER-positive tumours are metastatic at diagnosis, 20–40% of patients will ultimately develop recurrence at distant organs and such metastatic tumours are classified as incurable [36]. Therefore, new treatment strategies are urgently required to help patients that exhibit disease recurrence and metastasis.

One of the key findings from this work is the impact of CTSV on the expression of luminal biomarker, GATA3. GATA3 protein expression is a favourable prognostic indicator in ER-positive breast tumours, and numerous studies have reported that loss of GATA3 expression is associated with poor prognosis and propensity for tumour metastasis [18, 20, 23]. Research has revealed that GATA3 can regulate a number of genes associated with breast to lung metastasis [23], as well as impede breast cancer metastasis through the inhibition of EMT [22]. Whether CTSV can facilitate metastasis via suppression of GATA3 remains to be determined through the interrogation of in vivo model systems. Whilst previous studies have associated CTSV expression with distant metastasis [9] and postulated that CTSV is a liver-tropic gene in lung cancer [38], no studies have been undertaken as yet to determine the contribution of CTSV to the metastatic process. However, our observation that CTSV depleted cells also exhibit reduced eATP, which has been shown to contribute to metastasis and pre-metastatic events such as EMT, migration and invasion [35, 39], is suggestive that examining the impact of CTSV on metastasis is warranted.

Our results have identified that CTSV mediates GATA3 phosphorylation at Ser308, which facilitates GATA3 ubiquitination and subsequent degradation by the proteasome. Increased GATA3 turnover mediated by increased proteasomal degradation has previously been reported in breast cancer cells and T cells. Initial studies in T cells postulated that the C-terminal region of GATA3, between residues 261 and 315, is critical for proteasomal degradation [31], and further work in ER-positive breast cancer cells subsequently identified serine residue 308 as the critical site [29]. Research has suggested that examination of pSer308 GATA3 expression in breast cancer patients could be predictive of GATA3 loss, enabling stratification of GATA3 positive tumours, as those predicted to lose GATA3 expression would be expected to display a poorer prognosis. Therefore, it would be of interest to undertake immunohistochemical analysis, to determine if pSer308 GATA3 and CTSV expression correlates in clinical samples and be predictive of GATA3 loss, which would be associated with a worse prognosis.

The association between CTSV and the Akt-GSK-3β pathway has not previously been reported. However, Akt and GSK-3β have both previously been associated with GATA3, with Akt reported to facilitate GATA3 phosphorylation in T cells [40] and GSK-3β known to promote GATA3 degradation in ER-positive breast cancer cells. Activation of Akt is usually associated with a pro-tumour phenotype; however, recent work has suggested that elevated pAkt may be associated with a good prognosis in luminal A ER-positive breast cancer, particularly within the context of PIK3CA mutations [41].

The E3 ligase component FBXW7α has previously been reported to facilitate GATA3 degradation by the proteasome in a GSK-3β-dependent manner, with GSK-3β acknowledged as the predominant kinase in FBXW7α substrate recognition [42]. Akt phosphorylation of GSK-3β results in its inactivation via formation of an autoinhibitory pseudosubstrate and subsequent degradation by the proteasome [43, 44]. Therefore, elevated pAkt resulting in depleted GSK-3β expression would lead to reduced activity of FBXW7α, and accumulation of GATA3 expression, as is evident in our shCTSV cell line models. Inhibition of FBXW7α by the pseudophosphatase STYX has previously been illustrated [33]; in our models, STYX expression in CTSV-harbouring cells stabilised GATA3 protein expression, to levels similar as that observed in CTSV-depleted cells. Comparable effects were noted in both MCF-7 and ZR-75-1 cells, strengthening our hypothesis that FBXW7α is involved in CTSV-mediated GATA3 turnover.

Previous studies have identified that PI3K inhibition can reduce GATA3 protein expression in T cells and prostate carcinomas [34, 45]. Our observation that PI3K inhibition can attenuate the elevated GATA3 protein expression associated with shCTSV cells complements previous studies and suggests that the stabilisation of GATA3 protein expression in CTSV-depleted cells is manifested through PI3K-Akt activity. However, what remains to be determined is how CTSV depletion leads to further activation of the PI3K-Akt-GSK-3β pathway, resulting in GATA3 stabilisation. What is of particular interest is that the sustained oncogenic activation of Akt in the two cell lines examined here arise via different mechanisms. MCF-7 cells possess a hyperactivating PI3KCA mutation, whereas ZR-75-1 cells have a loss of PTEN [46]. Given that we have observed similar effects in both of these cell line models with PI3K inhibition is suggestive that CTSV functions upstream of PI3K-Akt to manipulate GATA3 protein expression. Due to the lack of PTEN in ZR-75-1 cells, it is unlikely that CTSV is mediating an effect via manipulation of PTEN, but it could have an impact on other negative regulators of the PI3K-Akt pathway such as SHIP2 or INPP4B. Furthermore, it is also plausible these effects could be mediated via the action of extracellular CTSV on receptor tyrosine kinases or G-protein coupled receptors, given that these receptors are the most common upstream activator of intracellular signalling pathways like PI3K-Akt. Evidence has shown that cysteine cathepsins including CTSV can be secreted from tumour cells [47], and recent publications have identified ectodomain shedding is undertaken by cathepsin family members to influence intracellular signalling pathways [48, 49]. Whilst our emphasis has been on the Akt-GSK-3β pathway, it is also possible that other kinases may facilitate the phosphorylation of GATA3, given the signalling intricacies of tumour cells, with PKA and p38 MAPK both previously identified as facilitating GATA3 phosphorylation [29, 50].

## Conclusions

In conclusion, we have identified that elevated CTSV expression is associated with reduced survival in ER-positive breast cancers. We have identified that CTSV promotes ER-positive breast cancer through facilitating tumour cell proliferation, invasion and suppressing the expression of GATA3. CTSV has not previously been associated with the Akt-GSK-3β-GATA3 pathway and whether it facilitates the degradation of other proteins by the proteasome remains to be elucidated. These results suggest that CTSV may represent a viable therapeutic target in ER-positive breast cancer; however, comprehensive analysis of in vivo models is necessary, particularly in relation to metastasis, which GATA3 has been shown to impair.

## Supplementary Information

**Additional file 1: Supplementary Fig. 1.** Representative images from clonogenic and invasion assay analysis. **a** Images from clonogenic assays performed with MCF-7 and ZR-75-1 cells. All images were captured and analysed using the Cell3 iMager neo. b Images from invasion assays performed with MCF-7 and ZR-75-1 cells. All images were captured on a Leica DM5500 fluorescent microscope at × 20 magnification.

**Additional file 2: Supplementary Fig. 2.** Densitometry analysis. a, b Densitometry analysis of GATA3 protein expression in MCF-7 and ZR-75-1 cells from 3 independent experiments, presented as mean relative densitometry units, with standard deviation.

**Additional file 3: Supplementary Fig. 3.** CTSV does not transcriptionally regulate GATA3 or impact RNA stability. a, b GATA3 mRNA expression was assessed by RQ-PCR in MCF-7 and ZR-75-1 shCTSV cells. c, d GATA3 mRNA expression was assessed by RQ-PCR following actinomycin D treatment (5 μg/μl for 8 h) in MCF-7 and ZR-75-1 shCTSV cells.

**Additional file 4: Supplementary Fig. 4.** Densitometry analysis. Densitometry analysis of GATA3 protein expression in MCF-7 and ZR-75-1 cells following Bortezomib treatment. Results are presented as mean relative densitometry units from 3 independent experiments, with standard deviation.

**Additional file 5: Supplementary Fig. 5.** Densitometry analysis. a, b Densitometry analysis of pThr308 Akt-1, pSer473 Akt-1, Akt-1, pSer9 GSK-3β, GSK-3β, pSer308 GATA3 and GATA3 protein expression in MCF-7 and ZR-75-1 cells following CTSV depletion and rescue experiments. c Densitometry analysis of GATA3 expression following transfection of STYX into MCF-7 and ZR-75-1 cells. d Densitometry analysis of GATA3 expression following treatment of MCF-7 and ZR-75-1 cells with Buparlisib (Bup). All results are presented as mean relative densitometry units from 3 independent experiments, with standard deviation.

## Data Availability

The datasets analysed during the current study are available via the KM Plotter repository [https://kmplot.com/analysis/index.php?p=service&cancer=breast] and via cBioPortal [https://www.cbioportal.org/study/summary?id=brca_tcga_pub].
