# Supplementary figures and images for: Cathepsin V suppresses GATA3 protein expression in luminal A breast cancer
Source: Breast Cancer Res. 2020 Dec 9;22:139. doi: 10.1186/s13058-020-01376-6 (PMC7726886; doi:10.1186/s13058-020-01376-6)

**A**

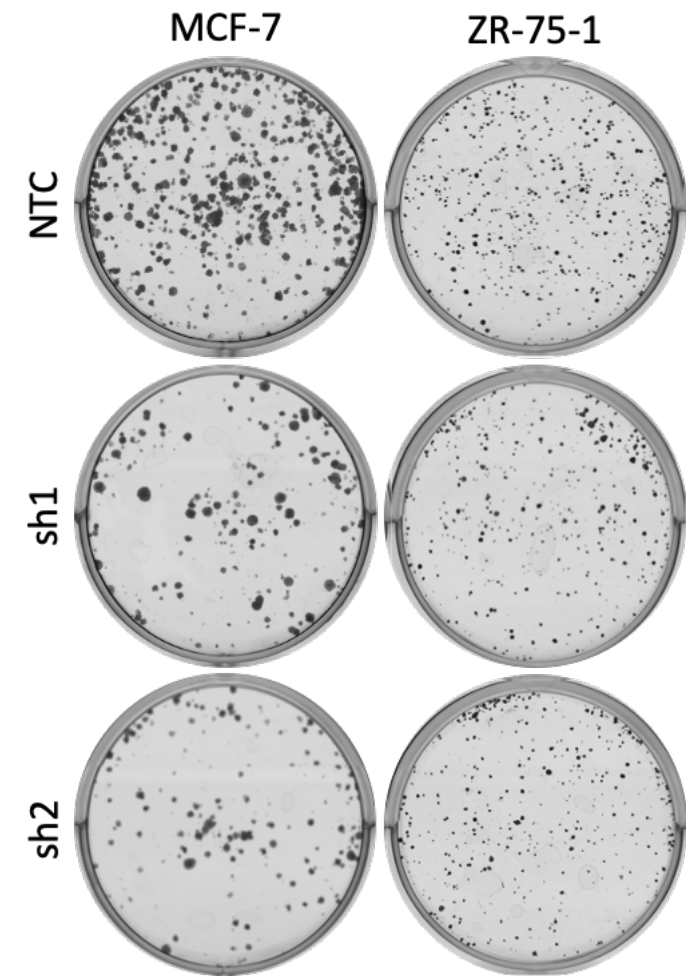

**B**

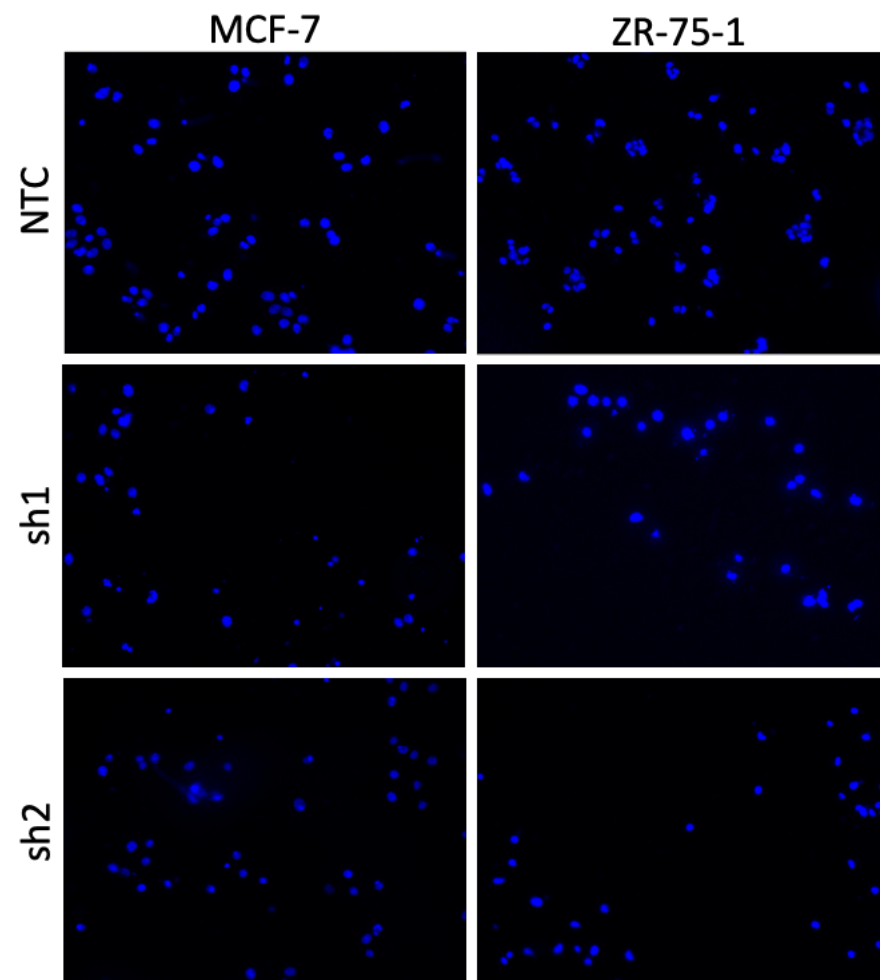

Supplement: Supplementary file 1 — Additional file 1: Supplementary Fig. 1. Representative images from clonogenic and invasion assay analysis. a Images from clonogenic assays performed with MCF-7 and ZR-75-1 cells. All images were captured and analysed using the Cell3 iMager neo. b Images from invasion assays performed with MCF-7 and ZR-75-1 cells. All images were captured on a Leica DM5500 fluorescent microscope at × 20 magnification. [file 13058_2020_1376_MOESM1_ESM.pdf]

**A**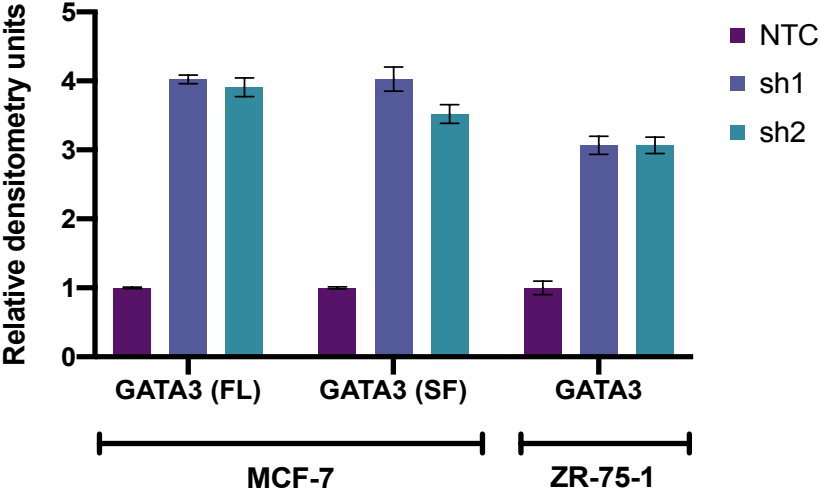**B**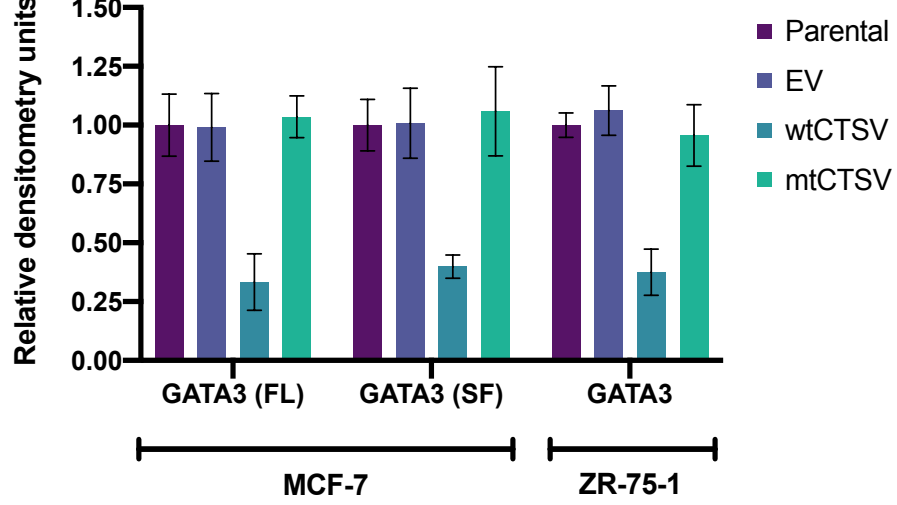

Supplement: Supplementary file 2 — Additional file 2: Supplementary Fig. 2. Densitometry analysis. a, b Densitometry analysis of GATA3 protein expression in MCF-7 and ZR-75-1 cells from 3 independent experiments, presented as mean relative densitometry units, with standard deviation. [file 13058_2020_1376_MOESM2_ESM.pdf]

**A****MCF-7**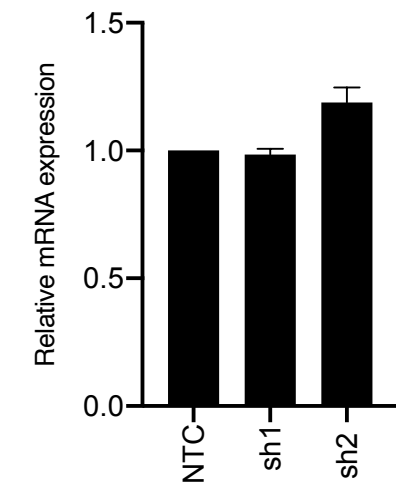**B****ZR-75-1**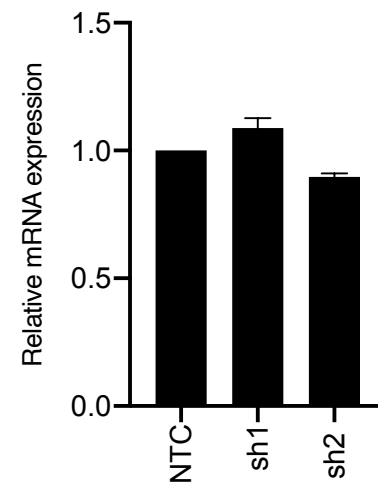**C****MCF-7**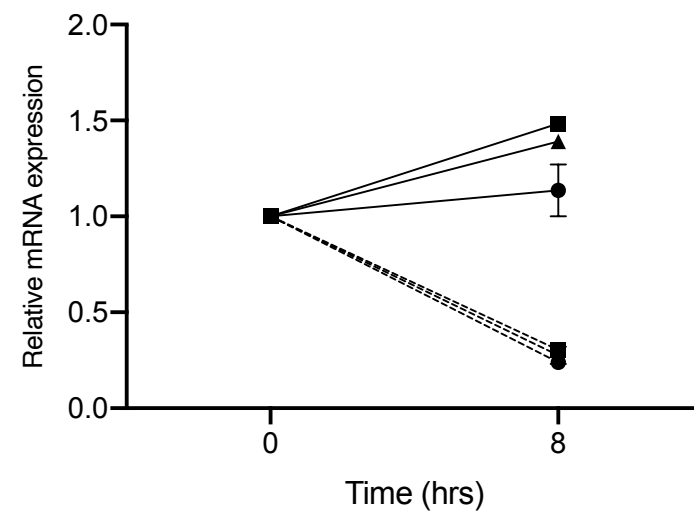

- NTC
- sh1
- ▲ sh2
- NTC+Act-D
- sh1+Act-D
- ▲--- sh2+Act-D

**D****ZR-75-1**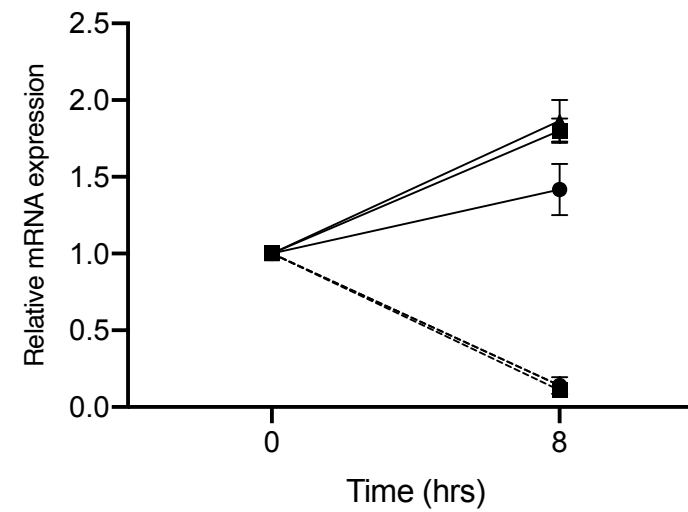

- NTC
- sh1
- ▲ sh2
- NTC+Act-D
- sh1+Act-D
- ▲--- sh2+Act-D

Supplement: Supplementary file 3 — Additional file 3: Supplementary Fig. 3. CTSV does not transcriptionally regulate GATA3 or impact RNA stability. a, b GATA3 mRNA expression was assessed by RQ-PCR in MCF-7 and ZR-75-1 shCTSV cells. c, d GATA3 mRNA expression was assessed by RQ-PCR following actinomycin D treatment (5 μg/μl for 8 h) in MCF-7 and ZR-75-1 shCTSV cells. [file 13058_2020_1376_MOESM3_ESM.pdf]

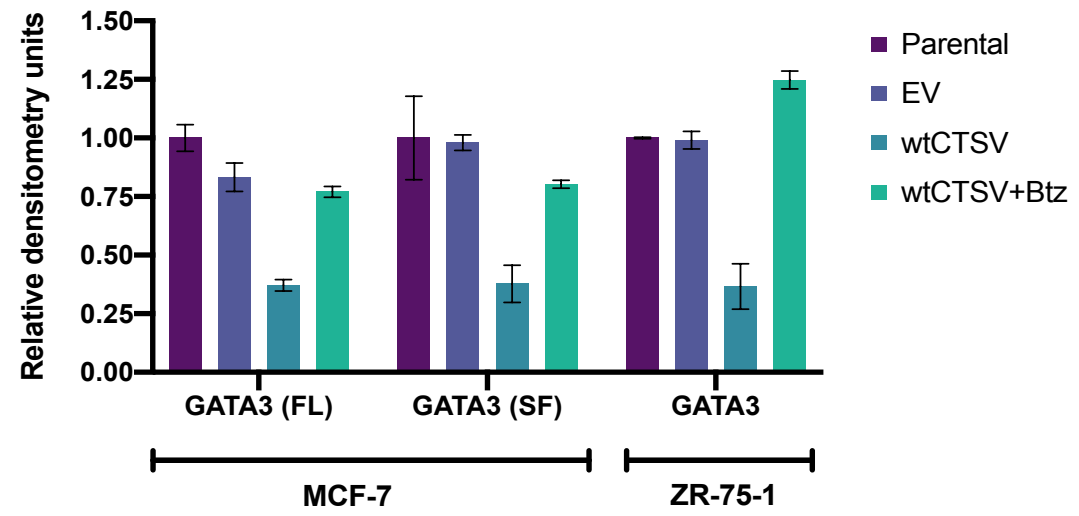

Supplement: Supplementary file 4 — Additional file 4: Supplementary Fig. 4. Densitometry analysis. Densitometry analysis of GATA3 protein expression in MCF-7 and ZR-75-1 cells following Bortezomib treatment. Results are presented as mean relative densitometry units from 3 independent experiments, with standard deviation. [file 13058_2020_1376_MOESM4_ESM.pdf]

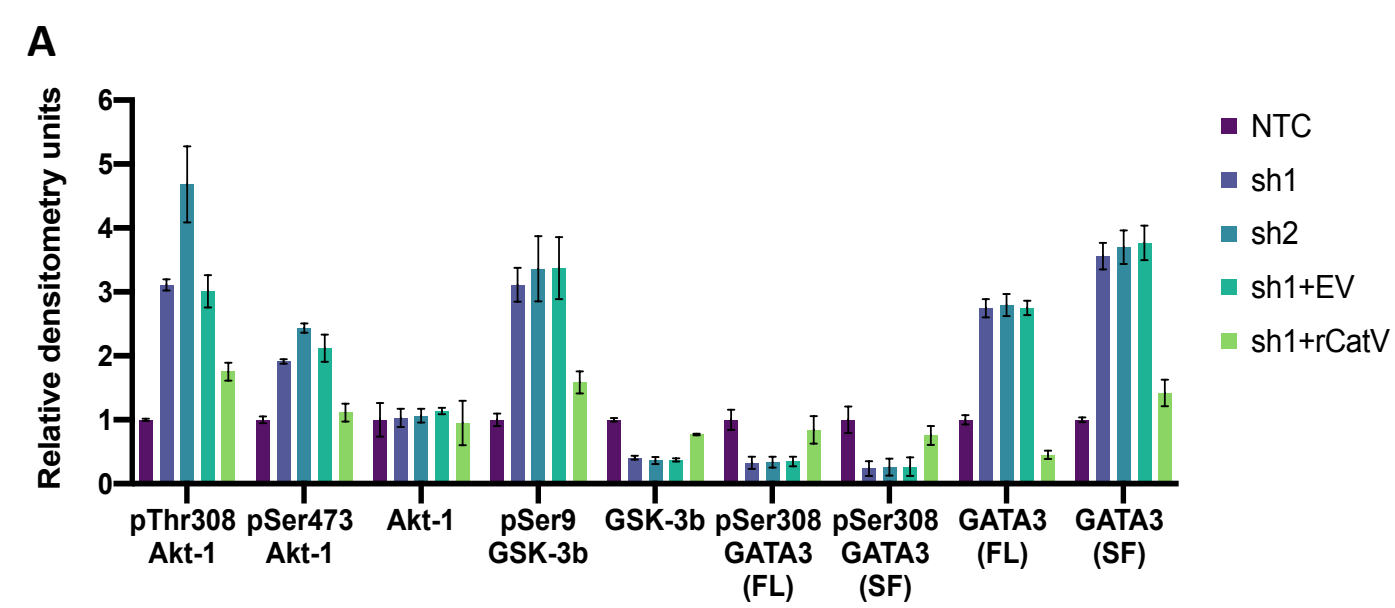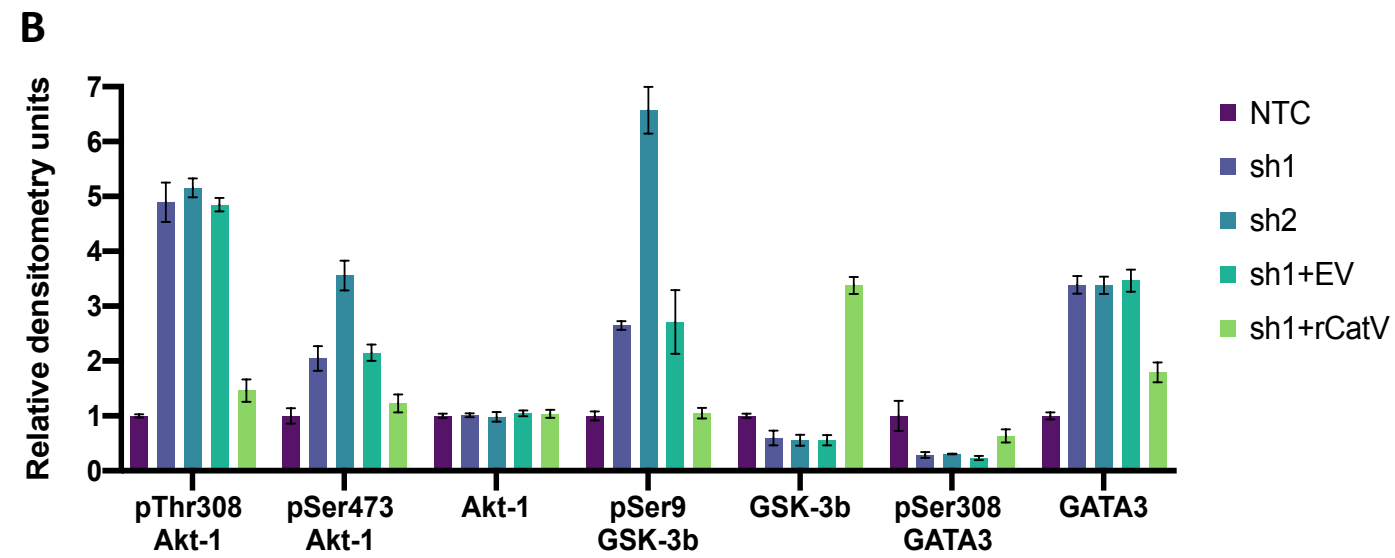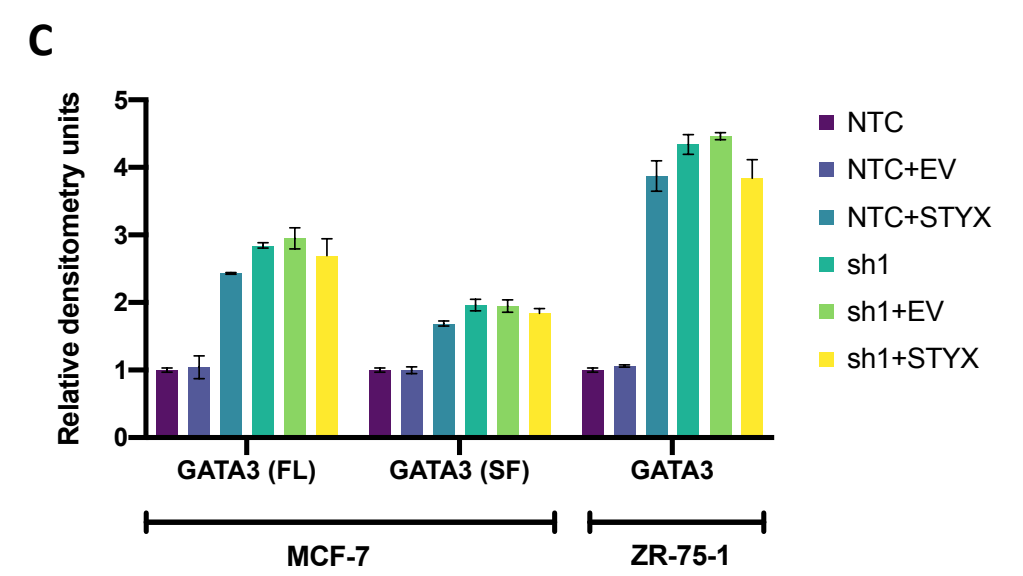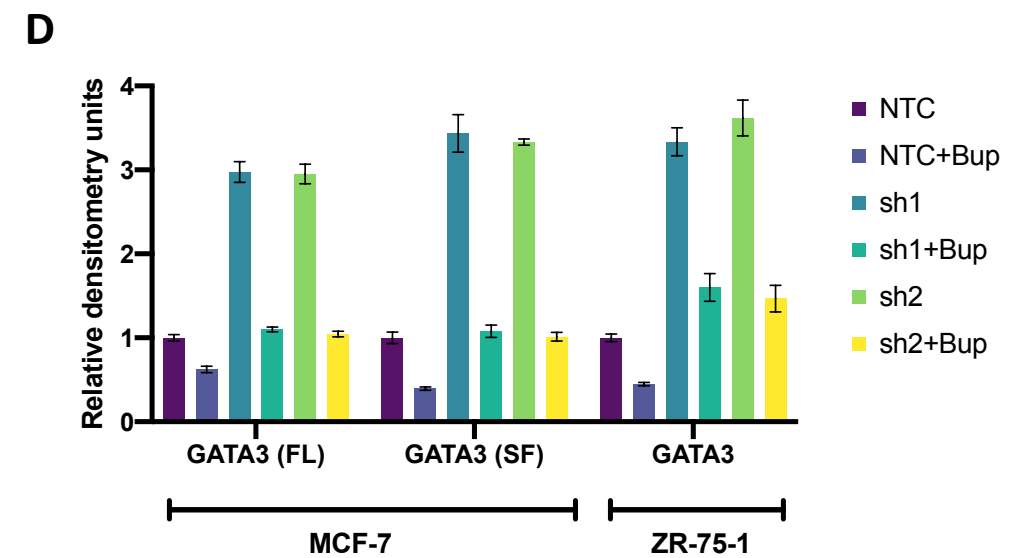

Supplement: Supplementary file 5 — Additional file 5: Supplementary Fig. 5. Densitometry analysis. a, b Densitometry analysis of pThr308 Akt-1, pSer473 Akt-1, Akt-1, pSer9 GSK-3β, GSK-3β, pSer308 GATA3 and GATA3 protein expression in MCF-7 and ZR-75-1 cells following CTSV depletion and rescue experiments. c Densitometry analysis of GATA3 expression following transfection of STYX into MCF-7 and ZR-75-1 cells. d Densitometry analysis of GATA3 expression following treatment of MCF-7 and ZR-75-1 cells with Buparlisib (Bup). All results are presented as mean relative densitometry units from 3 independent experiments, with standard deviation. [file 13058_2020_1376_MOESM5_ESM.pdf]
